# Theta and alpha oscillations may underlie improved attention and working memory in musically trained children

**DOI:** 10.1002/brb3.3517

**Published:** 2024-05-03

**Authors:** Leonie Kausel, F. Zamorano, P. Billeke, M. E. Sutherland, M. I. Alliende, J. Larrain‐Valenzuela, P. Soto‐Icaza, F. Aboitiz

**Affiliations:** ^1^ Centro de Estudios en Neurociencia Humana y Neuropsicología, Facultad de Psicología Universidad Diego Portales Santiago Chile; ^2^ Laboratorio de Neurociencia Social y Neuromodulación, Centro de Investigación en Complejidad Social (CICS), Facultad de Gobierno Universidad del Desarrollo Santiago Chile; ^3^ Centro Interdisciplinario de Neurociencias Pontificia Universidad Católica de Chile Santiago Chile; ^4^ Unidad de Imágenes Cuantitativas Avanzadas, Departamento de Imágenes Clínica Alemana de Santiago Santiago Chile; ^5^ Facultad de Ciencias para el Cuidado de la Salud Universidad San Sebastián Santiago Chile; ^6^ Laboratorio de Psiquiatría Traslacional Departamento de Psiquiatría Facultad de Medicina Universidad de Chile Santiago Chile; ^7^ Centro de Investigación en Complejidad Social (CICS), Facultad de Gobierno Universidad del Desarrollo Santiago Chile

**Keywords:** attention, musicians, neural oscillations, working memory

## Abstract

**Introduction**: Attention and working memory are key cognitive functions that allow us to select and maintain information in our mind for a short time, being essential for our daily life and, in particular, for learning and academic performance. It has been shown that musical training can improve working memory performance, but it is still unclear if and how the neural mechanisms of working memory and particularly attention are implicated in this process. In this work, we aimed to identify the oscillatory signature of bimodal attention and working memory that contributes to improved working memory in musically trained children.

**Materials and methods**: We recruited children with and without musical training and asked them to complete a bimodal (auditory/visual) attention and working memory task, whereas their brain activity was measured using electroencephalography. Behavioral, time–frequency, and source reconstruction analyses were made.

**Results**: Results showed that, overall, musically trained children performed better on the task than children without musical training. When comparing musically trained children with children without musical training, we found modulations in the alpha band pre‐stimuli onset and the beginning of stimuli onset in the frontal and parietal regions. These correlated with correct responses to the attended modality. Moreover, during the end phase of stimuli presentation, we found modulations correlating with correct responses independent of attention condition in the theta and alpha bands, in the left frontal and right parietal regions.

**Conclusions**: These results suggest that musically trained children have improved neuronal mechanisms for both attention allocation and memory encoding. Our results can be important for developing interventions for people with attention and working memory difficulties.

## Graphical abstract

Musical training improves attention and working memory in children. Musically trained children outperformed non‐trained children on a bimodal working memory task. Alpha band oscillation decreases are correlated with correct responses to the attended modality, whereas theta and alpha band oscillation increases are correlated with correct responses regardless of attention allocation.

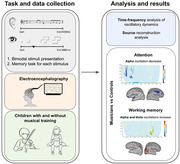

## 1 INTRODUCTION

Working memory (WM) is the key cognitive function that allows us to keep information in an accessible state for a short time and to use this information for the execution of cognitive processes. It is closely related to attention, which is the cognitive function that allows us to select the relevant stimuli for us at each moment. The ability to select, maintain, and manipulate information relevant to current behavioral goals is an essential part of our cognition (Paneri & Gregoriou, 2017) and is particularly relevant for learning and academic performance (Gathercole et al., 2004; Miranda et al., 2022). Furthermore, as we live in a multimodal world, where we encounter information that may not always be interconnected through various sensory modalities, it is crucial to understand how attention and WM function when exposed to various stimuli in different sensory modalities. Like other executive functions, attention and WM develop over time following the developmental trajectory of the brain, in particular, the development of the prefrontal cortex (Fiske & Holmboe, 2019; Reynolds & Romano, 2016).

One activity that has been shown to improve attention and WM is musical training (Kausel et al., 2020; Medina & Barraza, 2019; Talamini et al., 2016; Tierney et al., 2020). Even short periods of music instruction can improve WM (Guo et al., 2017). There is strong evidence for the improvement of auditory WM in musicians, whereas the positive impact of this kind of training on visual WM is less clear (Yurgil et al., 2020). Playing a musical instrument is a very complex task that involves various senses and motor dexterity, such as hearing what you play and reacting to it, producing the movements that are necessary to generate sound and inhibiting others, sometimes reading a score as well as looking at and communicating with others when playing in a group. Importantly, this practice has been shown to provoke changes in the structure and function of the brain (Schlaug, 2015). In sight of this, musical training has been proposed to be used as a model of brain plasticity (Herholz & Zatorre, 2012; Münte et al., 2002), which refers to the ability of the nervous system to change its activity in response to intrinsic or extrinsic stimuli by reorganizing its structure, functions, or connections. Even though the evidence of improved attention and WM in people with musical training is growing, the neural mechanisms that underlie these improvements are not well understood. A better understanding of these neural mechanisms is essential to better elucidate how brain plasticity and brain–behavior relationships work, which in turn is essential to develop better tools for the treatment of deficiency of these executive functions in mental disorders as well as to develop attention and WM interventions aimed at general educational practices (Klingberg, 2010).

Neural oscillations are one of the neurobiological mechanisms that underlie attention and WM. Attention has been related to modulations in alpha oscillations; in particular, an increase in alpha oscillations has been related to the inhibition of task‐irrelevant processes (Clayton et al., 2015), whereas a decrease in alpha oscillations has been associated with an adjustment of attentional engagement related to the adrenergic tone (Dahl et al., 2022). On the other hand, WM has been related to theta and alpha oscillations, which have been causally involved in this cognitive function (Riddle et al., 2020). In particular, an increase in frontal theta oscillations seems to be implicated in prioritizing information in WM (de Vries et al., 2020).

A recent review collected evidence for an impact of musicianship on neural oscillations involved in WM (Yurgil et al., 2020). One study showed that long‐range interhemispheric coherence of theta oscillations was greater in musicians than in nonmusicians during the encoding phase of a verbal memory task. Additionally, this theta coherence was positively associated with subsequent verbal WM performance (Cheung et al., 2017). Another study that investigated functional connectivity as measured by electroencephalography (EEG) in professional musicians found that during the more demanding verbal WM task, musicians outperformed nonmusicians and showed increased left‐hemispheric theta connectivity. Moreover, theta coherence was positively correlated with the number of years of music training (Dittinger et al., 2018). On the other hand, there is very scarce evidence of modulations in alpha activity associated with WM in musicians. One study that investigated prestimulus oscillatory activity related to WM performance in musicians and nonmusicians found that only musicians presented some characteristic features of alpha oscillations related to reaction time in the WM task (Klein et al., 2016). The exposed evidence indicates that musicianship could influence the oscillatory activity underlying WM, particularly in the theta and alpha frequencies. In contrast, it is not clear if and how musical training could affect oscillations related to attention processing and if these potential modulations could be related to better performance of musicians in WM tasks. Therefore, our understanding of the neural mechanisms underlying the improved performance in WM tasks in musically trained (MT) subjects still needs to be deepened at the level of neuronal activities, in particular to better discern how both attention and WM are impacted by the training and thus to gain knowledge of the phenomena that contribute to this improved behavioral performance. This is particularly relevant to understand in children, which is the period during which these executive functions mature and the brain is very plastic, and where musical training usually begins.

In a previously published magnetic resonance imaging (MRI) study of our group that reports results of the same study population and experimental paradigm presented in this article, we found that MT children were able to better attend and remember both auditory and visual stimuli than non‐MT (NMT) children on the bimodal (auditory/visual) attention and WM task, with MT children showing a higher activation of the frontoparietal control network and the phonological loop (Kausel et al., 2020). This suggests that both cognitive processes, attention and WM, are implicated in higher recalling of both auditory and visual stimuli. In this study, we research the oscillatory signatures of bimodal attention and WM as measured with EEG. We expected to find a better performance of the MT children in the task, in correlation with specific modulations in alpha and theta frequency bands.

## 2 MATERIALS AND METHODS

### 2.1 Participants

Forty healthy, right‐handed, Spanish‐speaking children aged 10–13, with normal hearing and normal or corrected‐to‐normal vision (Table 1), participated in our study after obtaining written informed consent from all children and their parents for a protocol approved by the Ethics Committee of the Pontificia Universidad Católica de Chile. Participants completed examinations from the end of 2016–2018. During their participation, they completed the Wechsler Intellectual Scale for Children (WISC III) (Wechsler, 1991) validated for the Chilean population (Ramírez & Rosas, 2007) and answered the Spanish version of the standardized Montreal Music History Questionnaire (Coffey et al., 2011), which inquired about their personal experience in music listening and performing. In the second and third sessions, participants solved the bimodal attention and WM paradigm, whereas their brain activity was either being measured with EEG or functional MRI (fMRI). The order of the experimental sessions was counterbalanced across participants. Importantly, when controlling for the order of the experimental sessions, results reported in this article do not change. Twenty MT children were recruited from different youth orchestras in Santiago, Chile. Inclusion criteria were as follows: playing a melodic instrument, having at least 2 years of instrumental lessons, practicing at least 2 h a week, and regularly playing in an orchestra or an ensemble. Six children played wind instruments (3 clarinets, 1 traverse flute, 1 horn, and 1 saxophone), and 14 played string instruments (12 violins, 1 viola, and 1 cello). Average musical training was 3.6 ± 1.5 years (range from 2 to 6 years), and all participants had studied music continuously since the onset of training. All children were trained based on more non‐aural strategies, could read and write musical scores, had individual or small group (two to three participants) instrumental lessons, and played in an orchestra, having rehearsals at least once a week over the last year. Twenty control (NMT) children were recruited from public schools in Santiago and had no additional musical training than the one provided in school curricula. In contrast to MT children, NMT children all declared to be unable to read or write musical scores.

**TABLE 1:** General demographics of the study population.

	Musically trained children	Control children	
***n***	17	19	
**Females**	9	11	
	**Mean ± SD**	**Mean ± SD**	***t*‐Value (*p* value)**
**Age (years)**	12.2 ± .8	12.0 ± 1.0	.58 (.28)
**IQ**	108.1 ± 8.1	105.2 ± 10.2	.90 (.18)
**Parental education**	4.0 ± 1.7	4.0 ± 1.6	.005 (.49)

*Note*: There were no significant differences between groups for age, IQ and parental education. IQ: intelligence coefficient.

Importantly, groups were matched for gender, age, intelligence coefficient (WISC III), and socioeconomic status evaluated with a basic approximation (educational level of both parents) (Table 1). For parental education, the highest, successfully completed education level of the parents was re‐coded into a measure reflecting level of education, ranging from 1 (incomplete middle school education) to 10 (complete PhD). The average of both parents was used (Liberatos et al., 1988). The guardian of one MT child did not provide father education, and the guardian of one control child did not provide parental education.

Results of the fMRI session are reported elsewhere (Kausel et al., 2020). In this article, we present the results of the EEG sessions. EEG recordings of four participants were excluded from the analysis because of excessive noise artifacts in the data acquisition. Finally, 17 MT children (9 female, mean age = 12.2 ± 0.8 years) and 19 NMT children (11 female, mean age = 12.0 ± 1.0 years) were included in the analysis (Table 1). Table 2 shows the musical training details of the MT children that were included in the final analysis.

**TABLE 2:** Characteristics of musical training in musically trained children.

Musically trained children (*n* = 17)		
*Group characteristics*	Mean ± SD	Range
Age at onset of musical training (years)	8.5 ± 1.9	4–11
Intensity of practice over the last year (h/week)	9.7 ± 5.2	2–21
Duration of musical training (years)	3.6 ± 1.5	2–6
***Type of musical instrument***	**Number of children**	
Strings	11	
Woodwinds	4	
Brass	2	

### 2.2 Experimental task

#### 2.2.1 Experimental paradigm

Participants solved the bimodal (auditory/visual) experimental paradigm adjusted from Kausel et al. (2020), whereas their brain activity was measured with EEG. This adjusted paradigm included 2 runs of 10 trials for each condition with unique stimuli. This paradigm is designed to measure attention and WM to stimuli in auditory and visual modality with attention being directed to only one or both stimuli at the same time (see below).

#### 2.2.2 Stimuli

**FIGURE 1:**
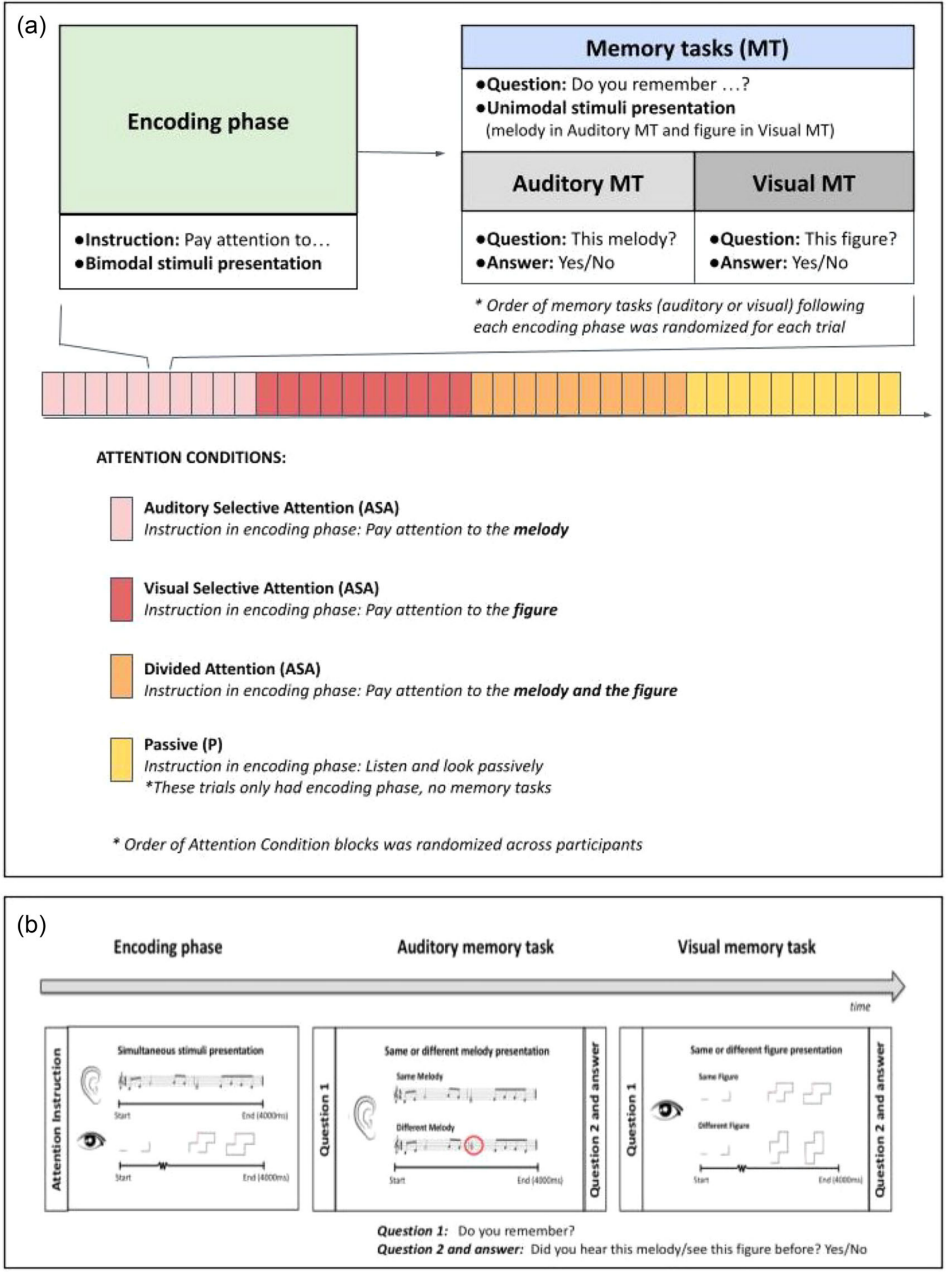
Experimental protocol. (A) Scheme of one run of the task. Each trial consisted of an encoding phase and the retrieval memory tasks. Each encoding phase presented bimodal stimuli (an auditory melody and a visual figure), preceded by an attention instruction which indicated to attend to either one or both stimuli at the same time. During the retrieval memory tasks, the auditory and the visual stimuli were tested separately with a yes/no question. The order of memory tasks was randomized for each trial. The passive condition only included the encoding phase. In each attention condition, 10 trials were presented as a block. Order of attention condition blocks was randomized across participants. (B) Trial structure that shows an example of the 4 s long stimuli that were presented in bimodal (encoding phase) or unimodal (memory tasks) manner.

Auditory (melodies) and visual (figures) stimuli were 4000 ms long. Melodies were in major tonalities and comprised pitches drawn from the Western musical scale centered around the mid‐range of the piano from F3 (175 Hz) to G6 (784 Hz), with half, quarter, and eighth notes. The melodies were presented in a tempo of 180 beats per minute (BPM), which implies that the duration of a quarter note is 333 ms. They were all in WAV format and were presented in a piano timbre. All melodies contained one chord, which had to be reported by button press during the auditory selective attention condition (ASA). The melodies were presented binaurally at a comfortable listening level for each subject through EEG‐compatible sound transmission headphones (Compumedics NeuroScan). Figures consisted of equally long nine black lines and one red line, which had to be reported by button press during visual selective attention condition (VSA). In order to “draw” each figure on a white background, individual shapes had the same starting point, and new lines were presented sequentially aligned either horizontally or vertically every 300 ms. An abstract shape formed by 10 consecutively incorporated lines was completed after 3000 ms and remained in view for 1000 ms. Children viewed the visual stimuli on a computer screen. A total of 160 melodies and figures were created. The auditory and visual stimuli started and stopped at exactly the same time, but the individual elements of the two stimuli never synchronized, because melodies and figures were not meant to be integrated into a unitary percept in order to avoid brain activity related to perceptual binding (Hipp et al., 2011). Stimuli were presented using Presentation Software (Neurobehavioral Systems) (Figure 1).

#### 2.2.3 Procedure

Each trial had two parts, the encoding phase and the memory retrieval phase (Figure 1). The encoding phase started with an instruction to pay attention to either (or both) the melody or figure. Then, two stimuli were simultaneously presented, including an abstract figure that was drawn on the screen over time (visual modality) and a melody (auditory modality). The attention instruction defined the three attention conditions, namely, ASA, VSA, and divided attention (DA) conditions. During the encoding phase, and in order to intensify selective attention, participants were asked to report the chord during ASA and the redline during VSA by button press. We also included one condition in which children were instructed to passively observe the stimuli (passive condition, P). These trials did not include the memory retrieval phase. During the memory retrieval phase, participants were asked to answer a same/different memory retrieval task for both the auditory (auditory memory task, AMT) and the visual (visual memory task, VMT) stimuli. Orders of the auditory and visual MTs after each encoding phase were randomized across trials. Answers to the memory retrieval tasks were reported via a button press. Better performance on the memory task for the attended stimuli was expected. Accuracy (correct responses) and reaction time of correct responses on the retrieval memory tasks were the behavioral outcome measures. The trials in the same attention condition were presented as a block. The order of attention condition blocks was randomized across participants. All conditions included unique stimuli and stimulus pairs presented during encoding phases were defined randomly for each subject. One run of the task had a duration of 13 min and included 40 trials, with 10 trials for each attention condition. All participants completed two runs of the task with unique stimuli, whereas their brain activity was measured with EEG.

#### 2.2.4 Data acquisition

The EEG registration was set up at the Laboratorio de Neurociencia Cognitiva y Evolutiva of the Medical Faculty of the Pontificia Universidad Católica de Chile. Participants were prepared for EEG and instructed to relax, keep still, and not blink during responses. EEG data were recorded continuously using a 40‐channel scalp electrode cap (Ag/AgCl) (NeuroScan Nuamps) in accordance with the international 10/20 system. All electrodes were referenced to the averaged recordings from the right and left mastoids during acquisition. Electrode impedance was maintained below 5 kΩ. Electrooculography (EOG) was recorded from electrodes placed above and below the left eye (monitor eye blinks or vertical eye movements) and lateral from the right and left eyes (monitor horizontal eye movements). EEG and EOG signals were recorded using the NeuroScan Nuamps amplifier, digitally high‐ and low‐pass filtered at 0.10 and 200 Hz, respectively, and sampled at 1000 Hz. After the EEG session, the position of the electrodes on the scalp of the participants was digitized with a Patriot digitizer (Polhemus).

### 2.3 Data analysis

#### 2.3.1 Behavioral data

Behavioral data were analyzed using RStudio (R Version 3.6.3). In order to understand the performance of MT‐ and NMT‐trained children on our task, accuracy, and reaction time for the responses given to the WM tasks were analyzed with a 2 × 2 × 2 mixed analysis of variance (ANOVA) to compare the main effects and interactions of groups (between‐subject factor: musicians, controls), attention focus (within‐subject factor: focus, non‐focus), and retrieval WM task (within‐subject factor: VMT, AMT). The attention focus factor grouped responses to trials where attention was directed to the retrieved stimuli as “focus,” and trials where attention was not directed to the retrieved stimuli as “non‐focus.” Whenever the assumption of sphericity was violated, the Greenhouse–Geisser correction for epsilon was applied. Interaction effects were further assessed with pairwise *t*‐tests. Bonferroni correction for multiple comparisons was applied where necessary. An alpha level of .05 was used for all statistical tests.

#### 2.3.2 Electroencephalography

Each participant completed two rounds of the task, completing 20 trials in each attention condition. EEG data were processed with the LAN Matlab toolbox (https://github.com/neurocics/LAN_current) also used in prior work (Billeke et al., 2020; Figueroa‐Vargas et al., 2020). In order to understand what was happening during the encoding of the stimuli that could explain the behavioral results, epochs encompassing an interval from 1 s prior to the onset of the stimuli to the 4 s duration of the stimuli were extracted for the encoding phase of each attention condition. EEG signals were preprocessed using an IIR Butterworth filter with a stopband frequency of 0.05 Hz, a passband frequency of 0.1 Hz, a stopband attenuation of 15 dB, and a passband ripple of 1 dB. Eye blinks were identified and removed from each dataset using independent component analysis. Other remaining artifacts (e.g., muscular artifacts) were detected using threshold criteria of the time series amplitude (>3 standard deviations) and amplitude of Fourier spectrum (>2 standard deviations of more than 20% of the spectrum [1–80 Hz]). Finally, a visual inspection of the row signals was performed in order to identify any remaining artifacts. The signal of noisy channels was interpolated by Spleninan interpolation implemented in EEGLab (Delorme & Makeig, 2004). Trials with remaining artifacts were eliminated from the analysis. Time–frequency analysis was performed using a 5‐cycle Morlet wavelet on the epochs. For all analyses, we used the dB of power related to the baseline (−1 to 0 s previous to stimulus presentation in the passive condition). We explored for differences between groups and conditions using the Wilcoxon test.

Time–frequency analysis was performed on the artifact‐free trials using a 5‐cycle Morlet wavelet transform from 1 to 45 Hz using 1 Hz of frequency resolution and 5 ms of temporal resolution. For the statistical analysis, we first modeled the power of the oscillatory activity with the general linear model per trial in each subject (first‐level analysis). From this, we obtained a 3D *t*‐value matrix (sensor, time, and frequency) for each regressor and subject.

The models were as follows:

Model 1:

$$\begin{array}{ccc} \mathrm{Power}\left(f,t\right) & = & b_{1}+b_{2}\mathrm{ASA}+b_{3}\mathrm{VSA}+b_{4}\mathrm{DA}+b_{5}\mathrm{ASAcAMT}\\ & & +b_{6}\mathrm{VSAcVMT}+b_{7}\mathrm{DAcAMTandVMT} \end{array}$$

Model 2:

$$\begin{array}{ccc} \mathrm{Power}\left(f,t\right) & = & b_{1}+b_{2}\mathrm{ASA}+b_{3}\mathrm{VSA}+b_{4}\mathrm{DA}+b_{5}\mathrm{ASAcAMT}\\ & & +b_{6}\mathrm{VSAcVMT}+b_{7}\mathrm{DAcAMTandVMT}+b_{8}\mathrm{ASAcVMT}\\ & & +b_{9}\mathrm{VSAcAMT} \end{array}$$

Models were applied to the epochs of the encoding phases described above. For Model 1, we included six dummy regressors, which correspond to the three active attention conditions (ASA, VSA, and DA) and the trials where participants later responded correctly to the memory task of the attended stimuli (ASAcAMT, VSAcVMT, and DAcAMTandVMT), where ASAcAMT is correct responses to AMT during ASA; VSAcVMT is correct responses to VMT during VSA; DAcAMTandVMT is correct responses to VMT and AMTs during DA. For the comparison between groups, we used the mean of ASAcAMT, VSAcVMT, and DAcAMTandVMT regressors per participant, in order to capture the common activity to correct responses to attended modality. In other words, as shown in the model specification, all encoding phase trials are included in the analysis (i.e., encoding phases followed by correct or incorrect answers), and the regressors of trials followed by correct responses to the attended modality allow us to isolate the activity that characterizes successful memory performance to attended stimuli.

For Model 2, we additionally included two dummy regressors, which correspond to the trials where participants later responded correctly to the memory task of the unattended stimuli (ASAcVMT and VSAcAMT), where ASAcVMT is correct responses to VMT during ASA; VSAcAMT is correct responses to AMT during VSA. For the comparison between groups, we used the mean of ASAcAMT, VSAcVMT, DAcAMTandVMT, ASAcVMT, and VSAcAMT regressors per participant, in order to capture the common activity that precedes correct responses irrespective of attention focus.

We then performed a Wilcoxon test to assess differences between groups at a second‐level analysis. To account for multiple comparisons in time–frequency charts, we implemented a cluster‐based permutation test (Maris & Oostenveld, 2007). This technique involved defining clusters of significant areas by pooling neighboring sites in the time–frequency chart that displayed the same effect (cluster threshold detection, CTD, uncorrected *p* < .05). The cluster‐level statistics were calculated by summing the statistics of all sites within the corresponding cluster, such as the *Z* value for the Wilcoxon test. To evaluate cluster‐level significance, we computed the permutation distribution of the largest cluster‐level statistics using group label permutations (for between‐subject analyses). For each permutation, the original statistical test was performed (i.e., the Wilcoxon test), and the resulting cluster‐level statistics of the largest cluster were used to establish the permutation distribution. After 1000 permutations, the proportion of elements in the permutation distribution greater than the cluster‐level statistics of the corresponding cluster was used to estimate the cluster‐level significance for each observed cluster.

Source reconstruction of the oscillatory activity was performed using the digitized position of the electrodes and the individual structural T1 scan (acquired during the MRI session). An individual tessellated cortical mesh was used as a brain model to estimate the current source distribution. We defined approximately 3 × 5000 sources constrained to the segmented cortical surface (three orthogonal sources at each spatial location) and computed a three‐layer boundary element conductivity model for the physical forward model. The neural current density time series at each elementary brain location was calculated using Brainstorm (Tadel et al., 2011). The latter was estimated by applying a weighted minimum norm estimate inverse solution with unconstrained dipole orientations in single trials.

## 3 RESULTS

### 3.1 Behavioral results

**FIGURE 2:**
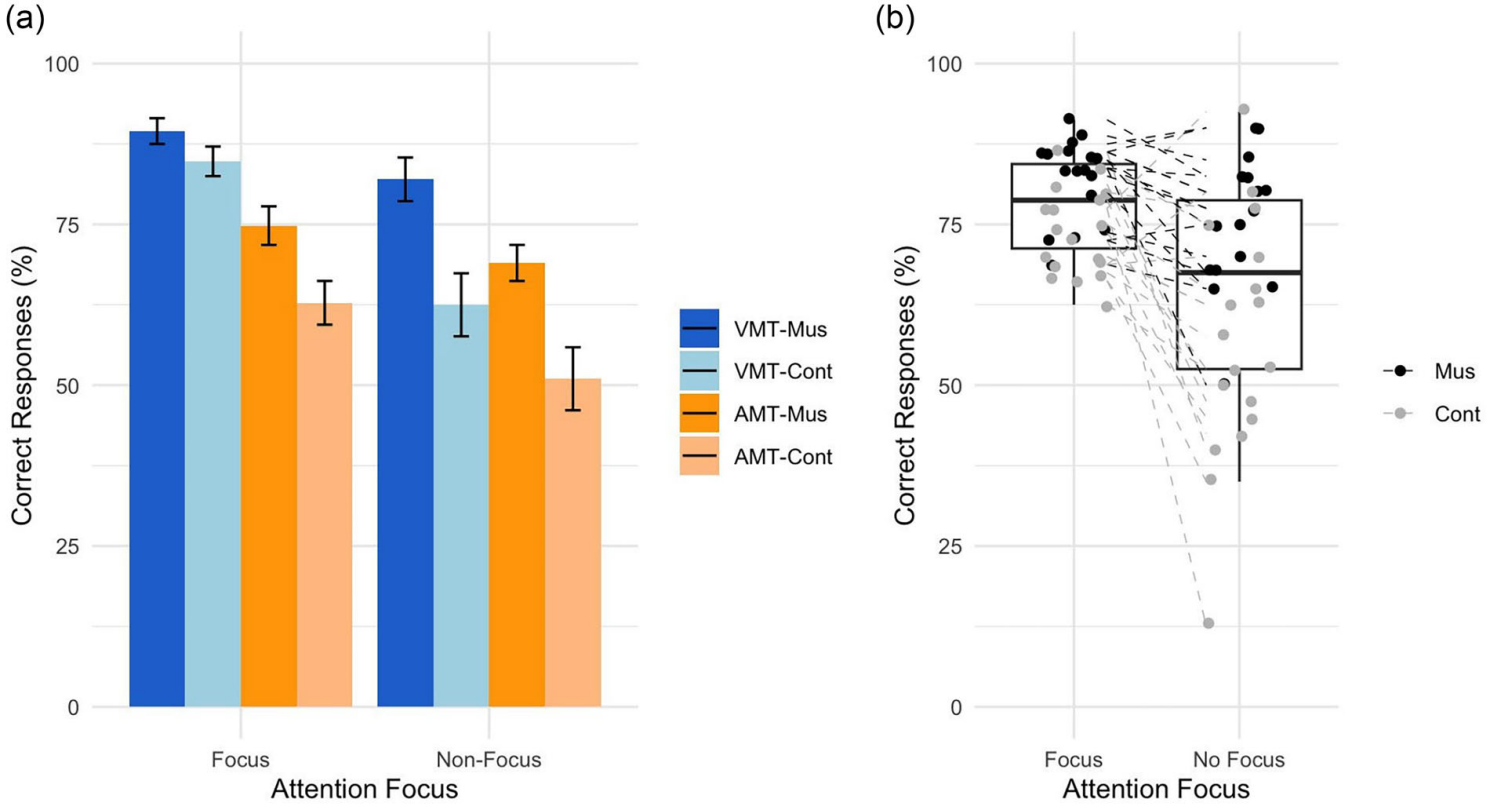
Accuracy in percentage of correct responses. (A) Accuracy of correct responses shown for attention focus, working memory (WM) task and group. Overall, musically trained children had significantly more correct responses than control children; children had significantly more correct responses when focusing on the stimuli then when not focusing on the stimuli; and children had significantly more correct responses in the visual than the auditory memory task (AMT). Error bars indicate standard error of the mean. VMT, visual memory task; Mus, musically trained children; Cont, control children. (B) Accuracy of correct responses by attention focus for each participant. Each point represents a subject (the mean correct responses of the subject for all WM tasks). The line represents the within‐subject effect between focus and non‐focus condition. The color represents the group of the subject (black for musically trained children—Mus; gray for control children—Cont).

Accuracy of correct responses is shown in Figure 2. Results were analyzed with a 2 × 2 × 2 mixed ANOVA with group, attention focus, and retrieval memory task as factors. The results for correct responses to WM tasks showed three significant main effects: first, a main effect of group (*F*(1,33) = 21.9, *p* = .000046, MSE = 12.8, ges = .18), showing that MT children had a significantly better performance on WM tasks than NMT children (Mus: mean = 15.98, SD = 2.7; Cont: mean = 13.6, SD = 4.0); second, a main effect of focus (*F*(1,33) = 20.7, *p* = .000068, MSE = 11.0, ges = .15), showing that children had a significantly better performance on WM tasks when attending to the retrieved stimuli than when not attending to the retrieved stimuli (focus: mean = 15.6, SD = 2.3; non‐focus: mean = 13.2, SD = 3.4); and third, a main effect of the retrieval WM task (*F*(1,33) = 37.3, *p* = .00000069, MSE = 9.0, ges = .21), showing that children had a significantly better performance on the VMT than on the AMT (VMT: mean = 16.4, SD = 2.4; AMT: mean = 13.2, SD = 2.9). No significant interaction effects were found. In summary, (i) MT children have an overall better performance on the memory tasks; (ii) all children remembered stimuli better when they focused attention on them than when they did not focus attention on them; and (iii) all children remembered visual stimuli better than auditory stimuli.

Behavioral results for the reaction time of correct responses showed no main effect or interaction effects among group, attention condition, and memory tasks. The overall mean reaction time was 834 ms (SD = 208 ms).

### 3.2 Time–frequency EEG results

**FIGURE 3:**
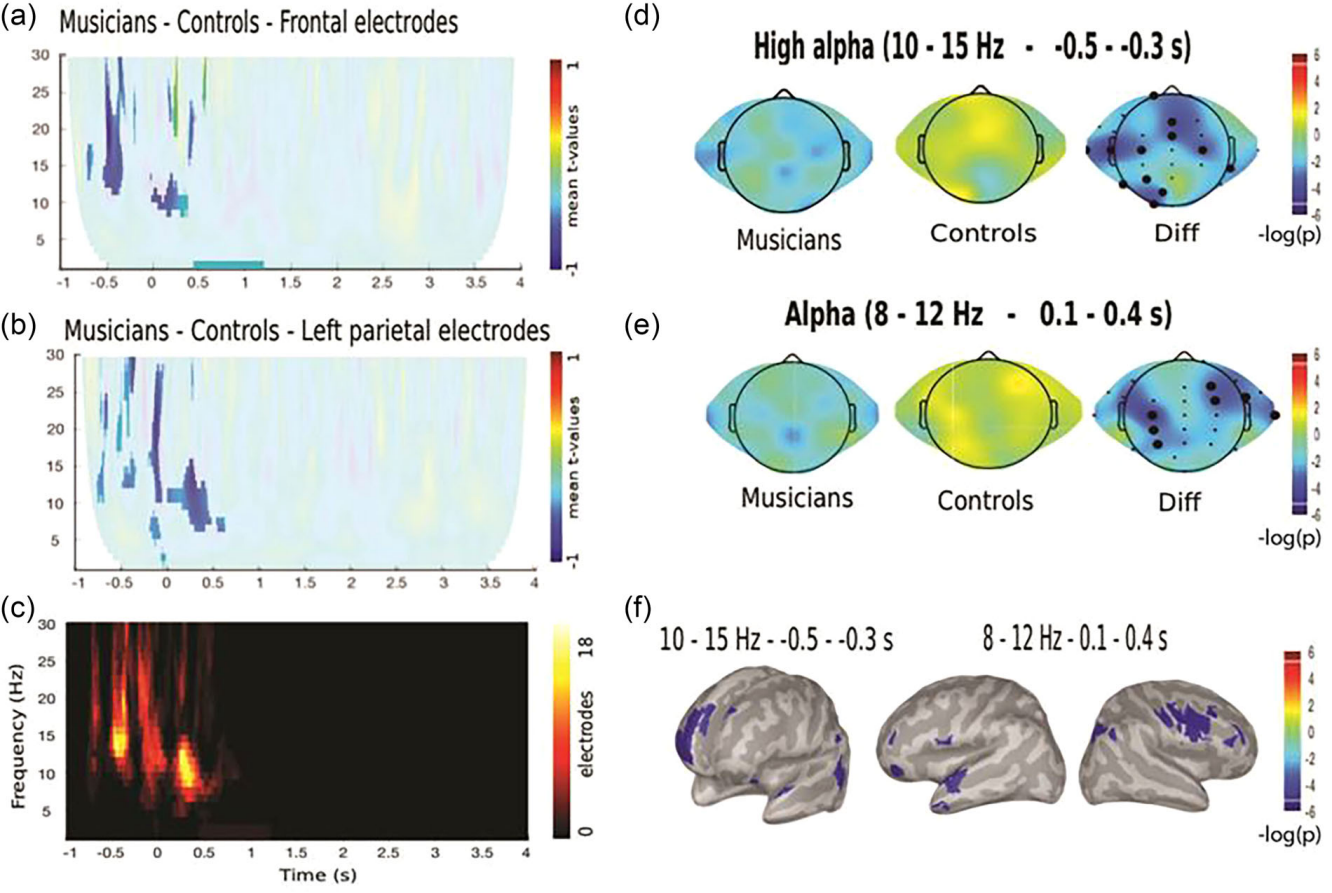
(A–E) Time–frequency analysis of the encoding phases of the correct auditory memory task (AMT) and visual memory task (VMT) when attending to the stimuli on the difference between musicians and controls. Color represents the mean *t*‐value of the single trial regressions per each subject given by the following equation (Model 1): Power(*f*,*t*) = *b*_1_ + *b*_2_ ASA + *b*_3_ VSA + *b*_4_ DA + *b*_5_ ASAcAMT + *b*_6_ VSAcVMT + *b*_7_ DAcAMTandVMT. Significant regions are highlighted (CBP test). (A and B) Time–frequency maps of the differences between groups regarding the aforementioned equation. (C) Significant clusters emerging for the contrasts shown in A and B. (D and E) Topographic distribution of alpha oscillations of the significant cluster in the time‐window specified in panel. (F) Source reconstruction of the differences between groups. The leftmost brain shows the difference in modulation of high alpha pre‐stimulus, and the middle and right brains show the difference of modulation of alpha poststimulus. Significant clusters (*p*_cluster_ < .01) and vertexes that survive vertex‐based correction are shown (FDR < .05). ASA, auditory selective attention; DA, divided attention; VSA, visual selective attention.

We studied the effect of attention focus and successful WM performance on the power of oscillatory brain activity during the encoding phase. As stimuli presented in the encoding phase where 4 s long (as detailed in Section 2), and in the time–frequency charts, the moment where stimuli presentation begins is time 0, when presenting the results, we refer to three points denominated: “pre‐stimuli onset,” which refers to differences that are present just before time 0; “beginning of stimuli onset,” which refers to differences that are present when the stimulus begins to be presented (time 0–0.5 s approx.); and “end of stimuli presentation,” which refers to differences that are present when the stimulus enters the ending moment of presentation (time 2.5–3.5 s approx.). Regarding successful performance by considering regressors that capture the difference between trials followed by correct and incorrect responses in the attended modality, we found a difference between groups that reflected a negative modulation in alpha activity around the stimulus onset (*p* < .001, Cluster‐based Permutation (CBP) test) (Model 1, Figure 3). This difference was driven by a decrease in the alpha frequency of the musicians’ group. The topographic distribution of this modulation was located in frontocentral and left parietal electrodes in pre‐stimuli onset (10–15 Hz, −0.5 to −0.3 s) and right frontal and left parietal electrodes at the beginning of stimulus onset period (8–12 Hz, 0.1–0.4 s). In the source analysis (described at the end of Section 2), we found that this modulation was located in the right frontopolar and left posterior temporoparietal areas (False Discovery Rate (FDR) *q* < .05) in pre‐stimuli onset and right inferior frontal junction and left anterior temporal areas (FDR *q* < .05) in at the beginning of stimulus onset.

**FIGURE 4:**
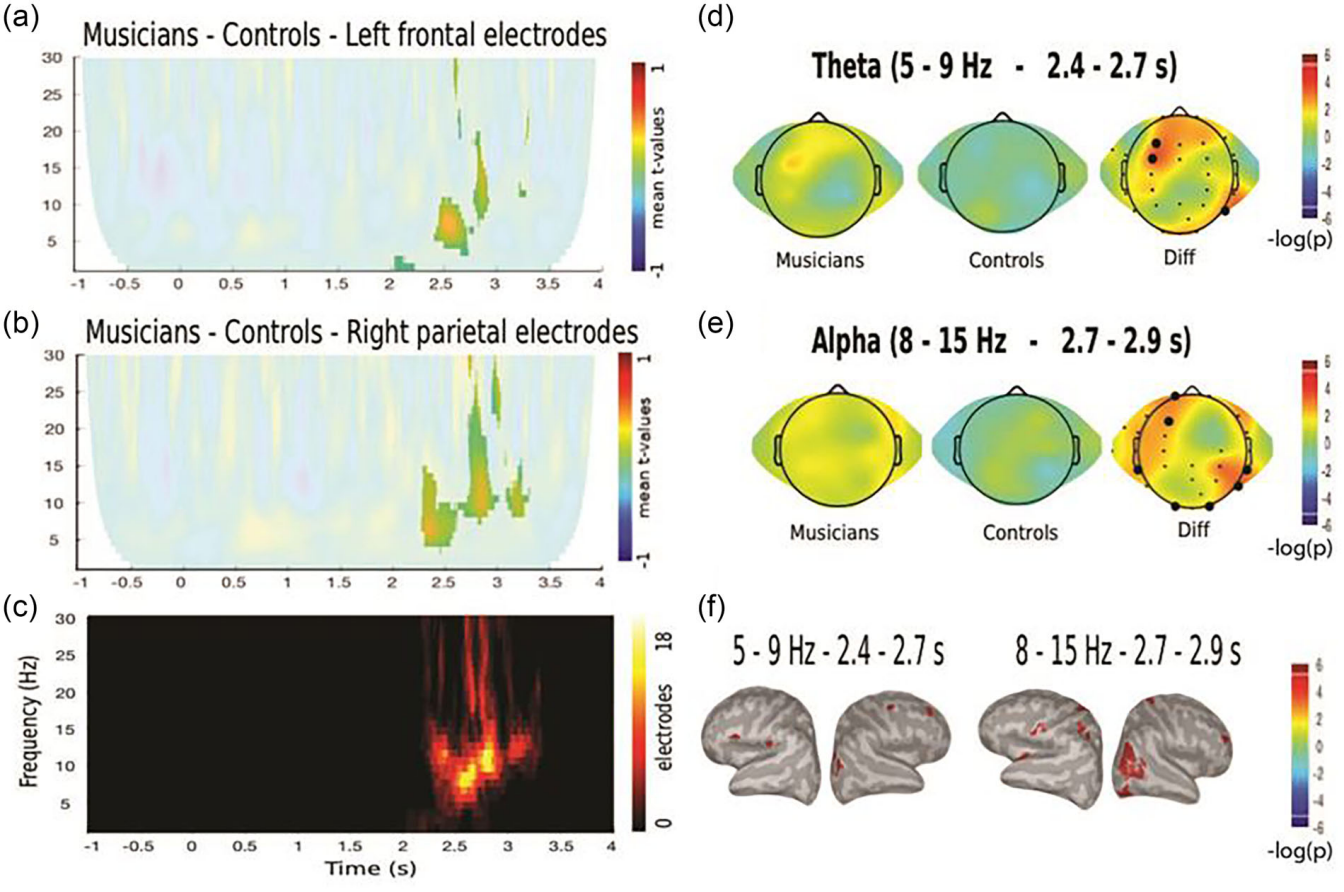
(A–E) Time–frequency analysis of correct auditory memory task (AMT) and visual memory task (VMT) in attended and unattended conditions on the difference between musicians and controls. Color represents the mean *t*‐value of the single trial regressions per each subject given by the following equation (Model 2): Power(*f*,*t*) = *b*_1_ + *b*_2_ ASA + *b*_3_ VSA + *b*_4_ DA + *b*_5_ ASAcAMT + *b*_6_ VSAcVMT + *b*_7_ DAcAMTandVMT + *b*_8_ ASAcVMT + *b*_9_ VSAcAMT. Significant regions are highlighted (CBP test). (A and B) Time–frequency maps of the differences between groups regarding the aforementioned equation. (C) Significant cluster emerging for the contrasts shown in A and B. (D and E) Topographic distribution of theta and alpha oscillations of the significant cluster in the timewindows specified in the figure. (F) Source reconstruction of the differences between groups. Both left brains show the difference of modulation of theta, whereas both right brains show the difference of modulation of alpha. Significant clusters (*p* < .01 cluster corrected) and vertexes that survive vertex‐based correction are shown (FDR *q* < .05). ASA, auditory selective attention; DA, divided attention; VSA, visual selective attention.

Next, we studied the oscillatory activity during the encoding phase associated with successful auditory or visual WM performance irrespective of attention focus. This includes the activity during the encoding phase that precedes correct responses in attended or unattended modality. We found differences between groups that showed an increase in theta and alpha activity in the ending period of the encoding phase (2.4–2.9 s) in the musicians’ group (cluster‐based permutation test, CTD *p* < .05, *p*_cluster_ < .01, Model 2, Figure 4). The topographic distribution of these modulations was located in the left frontal and right parietal electrodes. Source analysis showed that, for theta modulation, there is a bilateral distribution in frontoparietal areas (FDR *q* < .05), and, for alpha modulation, there is also a bilateral distribution in frontoparietal areas and right occipitotemporal areas (FDR *q* < .05).

## 4 DISCUSSION

In this research, we aimed to investigate if and how the neural oscillations that underlie attention and WM are related to a better performance of bimodal WM using musical training as a model. Children with and without musical training solved a bimodal attention and WM paradigm, whereas their brain activity was measured with EEG. Our results highlight three main findings. The first one is that MT children exhibit an overall better performance on the experimental paradigm than NMT children. The second and third findings are related to differences among groups of the oscillatory activity during the encoding phase associated with performance. On one hand, we found that MT children had a lower alpha power immediately pre‐ and beginning of stimuli onset than NMT children associated with correct responses to attended stimuli. The source of this decreased activity was localized to frontal and temporoparietal areas. On the other hand, we found that MT children had a higher theta and alpha power at the end of stimuli presentation than NMT children associated with correct responses independent of attention focus. The source of this activity was localized to frontal and parietal areas in the theta band, and frontal and occipitotemporal areas in the alpha band.

Our behavioral results showed that MT children overall had better performance on the task than NMT children. These results are in line with other research that has shown that playing a musical instrument can be related to improve auditory and visual WM in children (Bergman Nutley et al., 2014; Degé et al., 2011; Kausel et al., 2020). In fact, this improvement in WM in musicians has also been shown in adults (Criscuolo et al., 2019). Based on longitudinal studies that have found this effect, it is plausible to state that the improvement in the functioning of this executive function is due to training, and that it is not given by previous differences that could exist between the groups (Bergman Nutley et al., 2014; Degé et al., 2011). When comparing the results reported in this article from the EEG session to those obtained during the MRI session (Kausel et al., 2020), on one hand, we find the same result that shows that MT children overall have a better performance on the task than NMT children. Nevertheless, on the other hand, we did not find that MT children had a significantly higher performance than NMT children on the AMT when they were not paying attention to the auditory stimuli. We propose that this could have to do with the auditory environment in which the experiments occur. Children solved the task in a quiet laboratory during the EEG experiment, whereas they solved the task in the loud scanner during the fMRI experiment. The background noise of the scanner while not paying attention to the auditory stimuli could have attenuated the encoding of these stimuli in the NMT children, whereas MT children still were able to encode the auditory stimuli in this environment. This is in line with robust evidence that has shown that musicians have a higher encoding of speech in noisy environments (Coffey et al., 2017; Hennessy et al., 2022) that also seems to extend to better encoding of tonal stimuli in noise (Liang et al., 2016). If this effect could also be present when not paying attention to the auditory stimuli, such as what we suggest from our results, it remains to be elucidated.

In this study, we found two principal neurobiological findings regarding oscillatory differences related to performance between MT children and NMT children. One of our neurobiological findings was the brain activity associated with correct responses to the memory tasks in the attended modality during the encoding phase. Here we found that MT children had a lower alpha power immediately pre‐ and beginning of stimuli onset than NMT children, which was located at frontal and temporal areas. The decrease of alpha oscillations associated with correct responses in attended modality that occurs in MT children could indicate a stimulus‐specific information increase for both visual and auditory stimuli (Griffiths et al., 2019). This finding could be interpreted as elevating the signal of the stimulus above the noise generated by ongoing neuronal activity. In fact, evidence suggests that attention‐related modulations of alpha rhythms may provide a causal mechanism for the flexible selection of relevant information (Peylo et al., 2021) that take place in the frontoparietal network (Dahl et al., 2022). This is in line with our previous results showing a higher functioning of the frontoparietal control network in MT children while solving the bimodal experimental paradigm (Kausel et al., 2020). Both results seem to indicate that musical training could impact attention‐related mechanisms, even when this has to be considered carefully in the case of our study, because we cannot exclude that our participants could have had differences in the functioning of their attention networks before starting training.

Another neurobiological finding was the modulation that we found in theta and alpha oscillations associated with the correct responses to all the memory tasks independent of whether children were paying attention to the stimuli or not (i.e., independent of attention focus) during the encoding phase. There we found that MT children had a higher power than NMT children in both these frequency bands at about 2.5–2.9 s after the start of stimuli presentation, located at frontal and parietal areas for the theta oscillation and occipitotemporal areas for the alpha oscillation. Both theta and alpha activity are associated with WM retention. In particular, the increased power of the theta band could indicate a higher encoding capacity (Herweg et al., 2020) of the MT children. On the other hand, the higher power found in the alpha band could reflect that MT children have a better inhibition of further sensory input that will protect the memory of the current stimuli (Wianda & Ross, 2019). As stimuli were 4 s long and constructed over time, the timing of the observed differences therefore coincides with a larger memory load. Theta oscillations have been shown to increase with increased memory load (Fernández et al., 2021). In view of the presented results, we suggest that MT children could have better memory capacity (Talamini et al., 2017) in part due to the increased capacity of memory load encoding, even though this must be thoroughly considered in the case of our study, because we cannot dismiss the possibility that our participants could have possessed different memory‐encoding capacities prior to the starting of the training.

Our first neurobiological finding seems to reflect an attentional phenomenon and our second neurobiological finding seems to reflect a memory‐encoding phenomenon. This considering that the first finding shows modulations around stimuli onset considering correct trials to the actively attended stimuli, and the second finding shows modulations toward the end of stimuli presentation considering correct responses independent of attentional focus. Taking this evidence into account, we propose that the better performance of MT children on our paradigm seems to be driven by both reinforced attentional and memory‐encoding neurobiological mechanisms. This emphasizes the importance of both these cognitive functions in better WM performance and helps to disentangle if and how they contribute to obtain this achievement.

We would also like to highlight the fact that our results add to evidence that musical training could have an effect on domain‐general cognitive skills, such as attention and WM, which could be beneficial for the individual beyond musical activities (Román‐Caballero & Lupiáñez, 2022). This could be especially important to consider in educational settings. Taking into account proposals that consider cognition to emerge as an interaction among the brain, the body, and the environment (Newen et al., 2018), it seems to be a good idea to potentiate activities that are more integral in school curricula, activities that integrate the whole body and multiple sensory modalities with tools and social interactions, such as musical training. This said, it remains to be elucidated which musical training regimens and what training conditions result in the best transfer effects as well as investigate for whom the training effects could be the most useful.

This research allowed us to gain new insight into the neurobiological mechanisms underlying improved bimodal WM using musical training as a model. The results suggest that improved auditory and visual WM in MT children is associated with alpha and theta band modulations during different time points of the encoding phase, suggesting that both attention and WM neurobiological mechanisms might be impacted by training. Results reaffirm the notion that musical training is a valuable model to study brain plasticity and that it can be used to improve our understanding of how activities that can be completed in everyday life have an impact on the neurobiological mechanisms of key executive functions, such as attention and WM. Further systematic studies of brain activity during attention and WM tasks are needed to throw light on the neural bases of enhanced attention and WM abilities in musicians as compared to nonmusicians. Our results could be relevant for both education and public health policies. A better understanding of the mechanisms underlying improved attention and bimodal WM are essential for the development and design of more precise training programs and intervention strategies to enhance these capacities in people from the general population as well as people with diseases, such as neuropsychiatric patients who have deficits in these skills in auditory and visual modality, such as people with ADHD (Brydges et al., 2017; Wang et al., 2021), multiple sclerosis (Figueroa‐Vargas et al., 2020; Simani et al., 2023), schizophrenia (Green et al., 2000; Wood et al., 2006), and autism (Larrain‐Valenzuela et al., 2017; Lin et al., 2017). Finally, these insights can also inspire those working on the development of artificial intelligence systems that seek to emulate cognitive processes such as attention and WM to produce complex and integrative systems based on neurobiological findings (Lindsay, 2020; Macpherson et al., 2021).

## AUTHOR CONTRIBUTIONS

**Leonie Kausel**: Conceptualization; formal analysis; funding acquisition; investigation; methodology; project administration; software; writing—original draft; writing—review and editing. **F. Zamorano**: Conceptualization; funding acquisition; writing—review and editing. **F. Aboitiz**: Conceptualization; funding acquisition; writing—review and editing. **P. Billeke**: Formal analysis; funding acquisition; methodology; software; writing—review and editing. **M. E. Sutherland**: Conceptualization; resources; writing—review and editing. **M. I. Alliende**: Investigation; writing—review and editing. **J. Larrain‐Valenzuela**: Investigation; writing—review and editing. **P. Soto‐Icaza**: Writing—review and editing.

## CONFLICT OF INTEREST STATEMENT

The authors declare no conflicts of interest.

## FUNDING INFORMATION

ANID Chile, Regular FONDECYT 1210659, Regular FONDECYT 1211227, Regular FONDECYT 1190513, Postdoc FONDECYT 3190914, Iniciación FONDECYT 11230607

### PEER REVIEW

The peer review history for this article is available at https://publons.com/publon/10.1002/brb3.3517.

## Data Availability

The raw data supporting the conclusions of this article will be made available by the authors, without undue reservation, to any qualified researcher.
